# Association between duration of early empiric antibiotics and necrotizing enterocolitis and late-onset sepsis in preterm infants: a multicenter cohort study

**DOI:** 10.1007/s00431-022-04579-5

**Published:** 2022-08-04

**Authors:** Thomas H. Dierikx, Nancy Deianova, Jip Groen, Daniel C. Vijlbrief, Christian Hulzebos, Willem P. de Boode, Esther J. d’Haens, Veerle Cossey, Boris W. Kramer, Mirjam M. van Weissenbruch, Wouter J. de Jonge, Marc A. Benninga, Chris H. van den Akker, Anton H. van Kaam, Nanne K. H. de Boer, Douwe H. Visser, Hendrik J. Niemarkt, Tim G. J. de Meij

**Affiliations:** 1 Department of Pediatric Gastroenterology, Emma Children’s Hospital, Amsterdam UMC, location Vrije Universiteit Amsterdam, De Boelelaan 1117, room PK-1 Z 050, Amsterdam, 1081 HZ The Netherlands; 2 Amsterdam Gastroenterology Endocrinology Metabolism Research Institute, Amsterdam, the Netherlands; 3 Department of Neonatology, Wilhelmina Children’s Hospital, University Medical Center Utrecht, Utrecht, the Netherlands; 4 Division of Neonatology, Beatrix Children’s Hospital, University Medical Center Groningen, University of Groningen, Groningen, the Netherlands; 5 Department of Neonatology, Amalia Children’s Hospital, Radboud UMC, Radboud Institute for Health Sciences, Nijmegen, The Netherlands; 6 Neonatal Intensive Care Unit, Amalia Children’s Center, Isala, Zwolle, the Netherlands; 7 Department of Neonatology, University Hospitals Leuven, Leuven, Belgium; 8 Neonatal Intensive Care Unit, Department of Pediatrics, Maastricht UMC, University Maastricht, Maastricht, the Netherlands; 9 Department of Neonatology, Amsterdam UMC, Location University of Amsterdam, Amsterdam, the Netherlands; 10 Amsterdam Reproduction and Development Research Institute, Amsterdam, The Netherlands; 11 Tytgat Institute for Liver and Intestinal Research, Amsterdam Gastroenterology Endocrinology Metabolism Research Institute, Amsterdam UMC, location University of Amsterdam, Amsterdam, the Netherlands; 12 Department of Pediatric Gastroenterology, Amsterdam UMC, location University of Amsterdam, Amsterdam, the Netherlands; 13 Department of Gastroenterology and Hepatology, Amsterdam University Medical Centers, Vrije Universiteit Amsterdam, Amsterdam, the Netherlands; 14 Department of Neonatology, Máxima Medical Center, Veldhoven, the Netherlands

**Keywords:** Empirical antibiotics, Late-onset sepsis, Necrotizing enterocolitis, Preterm infant

## Abstract

The threshold to initiate empiric antibiotics for suspicion of early-onset sepsis (EOS) is low in preterm infants. Antibiotics’ effects on short-term outcomes have recently been debated. We aimed at exploring the extent of early empiric antibiotic exposure (EEAE) in preterm infants and the association between the duration of EEAE with necrotizing enterocolitis (NEC) and late-onset sepsis (LOS) within different EEAE groups. EEAE practice for suspicion of EOS was evaluated in all included infants (gestational age < 30 weeks) born in 9 centers in the Netherlands and Belgium between Oct. 2014 and Jan. 2019. EEAE association with NEC and LOS development was analyzed by multivariate regression. After excluding 56 EOS cases, 1259 infants were included. A total of 1122 infants (89.1%) were exposed to empirical antibiotics for the suspicion of EOS of whom 802 (63.7%) had short (≤ 72 h) and 320 (25.4%) prolonged EEAE (> 72 h). Infants with EEAE ≤ 72 h had a lower incidence of NEC compared to both infants without EEAE (adjusted odds ratio (aOR) 0.39; 95% confidence interval (CI) [0.19–0.80]; *p* = 0.01) and with prolonged EEAE (> 72 h) (aOR [95%CI]: 0.58 [0.35–0.96]; *p* = 0.03). With every additional day of EEAE, LOS incidence decreased (aOR [95%CI]: 0.90 [0.85–0.97]; *p* = 0.003).

*Conclusion*: Almost 90% of preterm infants who have negative blood culture results in the first 72 h of life are exposed to EEAE under suspicion of EOS. One-fourth has prolonged EEAE. Duration of EEAE was differently associated with NEC and LOS incidence. The effects of antibiotics, and potentially induced microbial dysbiosis related to development of NEC and LOS, should further be explored.

**Table:** 

**What is Known:** *• Preterm infants often receive antibiotics empirically directly after birth for suspicion of early-onset sepsis.* *• The effects of the duration of early empirical antibiotic exposure on the risk for necrotizing enterocolitis and late-onset sepsis are debated.*	
**What is New:** *• Almost 90% of preterm infants with a gestational age below 30 weeks are exposed to antibiotics empirically after birth despite negative culture results. In a quarter of these culture-negative infants, empirical antibiotics are prolonged.* *• A short course of empirical antibiotics (≤72h) is associated with decreased odds for necrotizing enterocolitis compared to both prolonged (>72h) or no empirical antibiotics after birth. Furthermore, every additional day of empirical antibiotic exposure is associated with decreased risk for late-onset sepsis in the first month of life.*

**Supplementary Information:**

The online version contains supplementary material available at 10.1007/s00431-022-04579-5.

## Introduction

Neonatal sepsis remains one of the leading causes of morbidity and mortality at the neonatal intensive care unit (NICU) [1]. Given the high burden associated with delayed treatment of early-onset sepsis (EOS), threshold for empiric initiation of antibiotics is low in preterm infants [2]. Consequently, over 75% of very low birth weight (VLBW; birth weight < 1500 g) infants are empirically exposed to antibiotics [3]. Empirical therapy is usually discontinued upon negative blood culture results after 48–72 h. However, as blood culture has low sensitivity, the course is often prolonged out of fear of undertreating clinical sepsis [2, 4].

Potential adverse effects of antibiotic exposure include antibiotic resistance and dysregulation of microbial gut colonization by decreasing the diversity and promoting overgrowth of potential pathogens [5]. Specifically at neonatal age, early empiric antibiotic exposure (EEAE) has been suggested to increase the risk of long-term adverse effects, such as development of metabolic and auto-immune disorders [5]. In the short term, it has been demonstrated in VLBW infants that every additional day of antibiotic exposure is associated with worse composite outcomes of multiple adverse events, including necrotizing enterocolitis (NEC) and late-onset sepsis (LOS) [6]. However, these findings have recently been questioned by observational and animal model studies, suggesting a mitigating effect of antibiotics on NEC [7, 8]. In murine models, antibiotics decrease bloodstream infections, potentially by delaying colonization, lowering the bacterial load at the level of the intestinal mucosa and the load of invasive microorganisms at the epithelial border [9].

This hypothesis is supported by two recent case–control studies performed by our group, showing that antibiotic exposure was associated with decreased odds of gram-positive LOS and, when initiated directly postpartum, with decreased odds of NEC [10, 11]. Neither study, however, focused specifically on EEAE for EOS suspicion and both were prone to confounding by indication, as antibiotic treatment and extension thereof could depend on clinical factors, which are also associated with NEC and LOS.

In the current larger multicenter cohort study, we aim to explore clinical characteristics associated with (prolongation of) EEAE and investigate the association between the duration of EEAE with NEC and LOS.

## Materials and methods

### Study design and participants

This study was embedded in an ongoing prospective multicenter preterm cohort study in nine participating NICUs in the Netherlands^1^ and Belgium,^2^ with the primary objective of identifying novel non-invasive biomarkers, as well as clinical risk factors, for LOS and NEC in the first 28 days of life [12]. Consequently, included participants have, in part, been described in previous case–control studies investigating fecal biomarkers and a wide range of risk factors for LOS and NEC [10, 11]. In our current study, we included all infants born before 30 weeks of gestation between October 2014 and July 2019 whose parents provided informed consent (Ethical Board permission A2020.190). Antibiotics for risk or suspicion of EOS were started by the attending physician in standard dosage and administered parenterally, according to the NICE guideline on *Antibiotics for early-onset neonatal infection* [13]. None of the participating centers routinely prescribed probiotics in the study period.

We excluded infants with major congenital malformations, including gastrointestinal malformation, such as anal or intestinal atresia and Hirschsprung’s disease [10, 11]. Additionally, in accordance with previous research, we excluded infants with culture-proven EOS and infants demised in the first week of life, irrespective of the cause of death [6, 14, 15]. Infants with culture-proven EOS were excluded since they require prolonged treatment with antibiotics, thus not being treated empirically. Finally, inaccessibility to patient record data on antibiotic exposure and morbidity was an additional exclusion criterion.

### Definitions

EEAE was defined as antibiotic exposure started within the first 72 h of life under the suspicion of EOS, but in the absence of a positive blood and, if applicable, cerebrospinal fluid culture. The duration was counted per started 24 h. Common antibiotic practice per center for suspicion of EOS with included number of participants is presented in Table S1. Subjects were categorized based on EEAE duration: (1) no EEAE; (2) short EEAE (≤ 72 h); or (3) prolonged EEAE (> 72 h). The cut-off point of 72 h was chosen in agreement with common clinical practice, where empiric antibiotic therapy is often discontinued within 48–72 h in case clinical and biochemical correlates for sepsis are missing [16].

Infants were classified as NEC cases, when diagnosed with NEC stage IIA or higher, according to the modified Bell’s staging criteria [17]. All infants with NEC were independently reviewed by two experts (TM, HN) for classification. In case of discrepancy, infants were reevaluated until an agreement was reached. All neonatal LOS episodes, defined as blood culture–proven sepsis with onset beyond the first 72 h and within the first 28 days, were analyzed and classified (Supplementary Table S2) [18, 19]. Infants could be classified as both NEC and LOS cases if they met the criteria for both.

Feeding practice was subcategorized as done previously, consisting of three categories: (1) human milk (HM), either own mother’s milk (MM) or donor milk (DM), (2) formula feeding (FM), (3) combination of HM and FM (Table S2) [11]. The highest C-reactive protein level within 72 h after birth was recorded. Inotropic medication and type of ventilation support were registered between 48 and 72 h after birth, as the decision whether to prolong empirical antibiotics is made at that moment. Standard demographic and clinical data were collected. Additional definitions of clinical and demographic characteristics are depicted in Table S2.

### Statistical analysis

Statistical analyses were conducted using the Statistical Package for Social Sciences (SPSS) version 26.0 (IBM, Armonk, NY, USA). Continuous demographic and clinical characteristics were depicted, depending on normality, as either mean and standard deviation (SD) or median and interquartile range [IQR] for the three groups of interest: (1) no EEAE, (2) short (≤ 72 h), and (3) prolonged (> 72 h) EEAE. Where appropriate, continuous data were analyzed by parametric one-way ANOVA, or non-parametric Kruskal–Wallis tests. The normal distribution of continuous data was assessed visually. Categorical data were analyzed by Pearson’s chi-squared test. Two-sided *p*-values of < 0.05 were considered statistically significant.

Associations between EEAE and incidence of NEC and LOS were analyzed by univariate and multivariate logistic regression methods with EEAE as a dichotomous variable (unexposed versus exposed infants). Secondly, the duration of EEAE was analyzed both as a categorical variable (no EEAE vs. short (≤ 72 h) vs. prolonged (> 72 h EEAE), and as a continuous variable (EEAE in number of calendar days).

In the multivariate models, odds ratios (ORs) were adjusted for confounding variables previously associated with NEC and LOS development [11, 20]: center of birth, gestational age, birth weight percentiles, gender, mode of delivery, invasive ventilation and/or inotropic medication use at day two of life, and type of enteral feeding. For LOS, a 5-min Apgar score and duration of parenteral feeding were added. Results from the logistic regression were reported as OR and adjusted OR (aOR), along with the respective 95% confidence interval (95%CI). Subgroup analyses for coagulase-negative staphylococcus (CoNS) and non-CoNS sepsis were additionally performed.

A post hoc uni- and multivariate analysis was performed to assess odds for LOS and non-CoNS LOS after exclusion of all LOS cases who were diagnosed before the postnatal age of 7 days. Although the most common definition of LOS is sepsis with onset ≥ 72 h of life, some clinicians, as well as several studies, define LOS as sepsis beyond the first week of life [19, 21]. With this analysis, we aimed at ensuring the comparability of our methods with those studies.

## Results

**Fig. 1:**
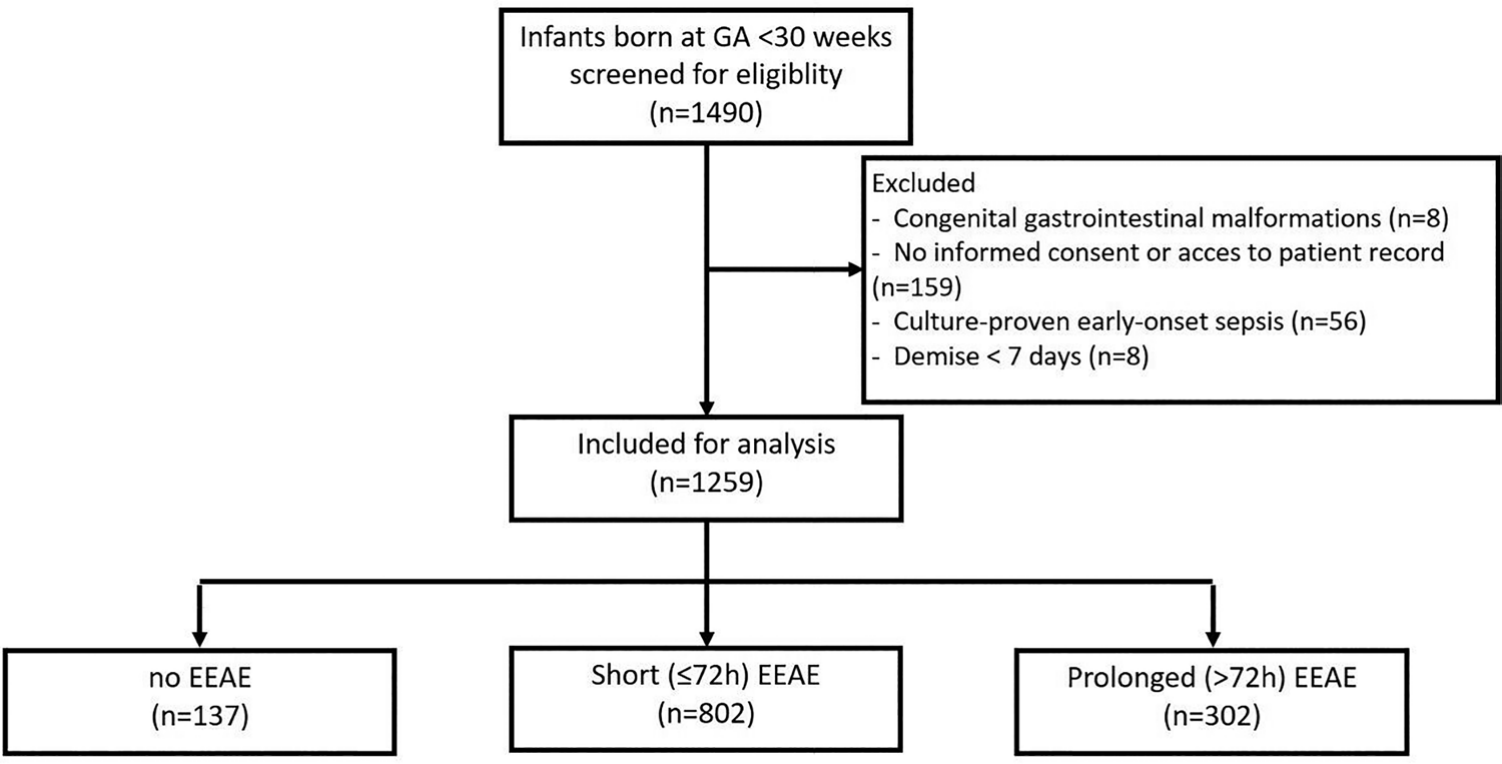
Flow chart of patient inclusions. EEAE: early empiric antibiotic exposure; GA: gestational age; h: hours

A total of 1490 infants born before 30 weeks of gestation were screened for eligibility between October 2014 and January 2019, of whom 231 were excluded. The main reasons for exclusion were lack of informed consent (*n* = 159) and culture-proven EOS (*n* = 56). Additional motives for exclusion are depicted in Fig. 1.

Of the 1259 included infants with negative blood culture results from the first 72 h of life, 1122 (89%) had EEAE for the suspicion of EOS, of whom 802 (64%) had short EEAE (≤ 72 h) and 320 (25%) prolonged EEAE (Fig. 1). Prolonged EAEE ranged between 19 and 44%, depending on the center of birth (Table S1).

Baseline characteristics are depicted in Table 1. Infants without EEAE were more often born by caesarean section and were smaller for gestational age (SGA), while infants with prolonged EEAE were invasively ventilated, needed inotropic medication, and had an increased CRP level (≥ 10 mg/dl) more often than the other groups.

**Table 1:** Demographic and clinical characteristics of all subjects categorized according to early empiric antibiotic exposure

	*No EEAE (n* = *137)*	*Short EEAE (n* = *802)*	*Prolonged EEAE (n* = *320)*	*p-value*
Gestational age, *weeks* + *days* (median [IQR])	28 + 6 [27 + 6–29 + 3]	27 + 6 [26 + 1–28 + 6]	27 + 1 [25 + 6–28 + 4]	< 0.001
Birth weight, *gram*, mean (SD)	1001 (280)	1055 (262)	940 (261)	< 0.001
Birthweight, *z-score*, mean (SD)	− 0.48 (0.94)	0.24 (0.80)	− 0.02 (0.90)	< 0.001
SGA, *n* (%)	27 (20)	41 (5)	31 (10)	< 0.001
Gender, *female*, *n* (%)	80 (58)	364 (46)	143 (45)	0.02
Delivery mode, *vaginal*, *n* (%)	14 (10)	439 (55)	145 (46)	< 0.001
Singleton, *n* (%)	110 (80)	534 (66)	210 (66)	0.002
Invasive ventilation at 48–72 h of life, *n* (%)	16 (12)	168 (21)	149 (47)	< 0.001
Inotropic medication at 48–72 h of life, *n* (%)	0 (0)	25 (3)	37 (12)	< 0.001
Enteral feeding type				0.01
Human milk, *n* (%) Formula milk, *n* (%) Combination, *n* (%)	80 (65) 16 (13) 28 (23)	562 (74) 100 (13) 101 (13)	232 (78) 22 (7) 43 (14)	
Parental feeding, *days* (median [IQR])	9 [6–11]	9 [7–11]	10 [8–11]	< 0.001
EEAE duration, *days* (median (IQR])	N/A	3 [2–3]	7 [6–8]	< 0.001
Highest CRP within first 72 h of life				< 0.001
≥ 10 mg/L, *n* (%) missing values, n (%)	1 (1%) 58 (42%)	37 (5%) 78 (10%)	103 (32%) 35 (11%)	
Age of NEC onset, *days* (median [IQR])	11 [9–20]	13 [9–20]	14 [10–18]	0.84
Age of LOS onset, *days* (median [IQR])	6 [4–10]	9 [6–12]	11 [9–15]	< 0.001

Data are summarized as mean and standard deviation (SD) or number and percentage (%), unless stated otherwise

*AB* antibiotics, *CRP* C-reactive protein, *EEAE* early empiric antibiotic exposure, *GA* gestational age, *LOS* late-onset sepsis, *N/A* not applicable, *NEC* necrotizing enterocolitis, *PPROM* premature prolonged rupture of membranes, *NICU* neonatal intensive care unit, *SGA* small for gestational age

**Fig. 2:**
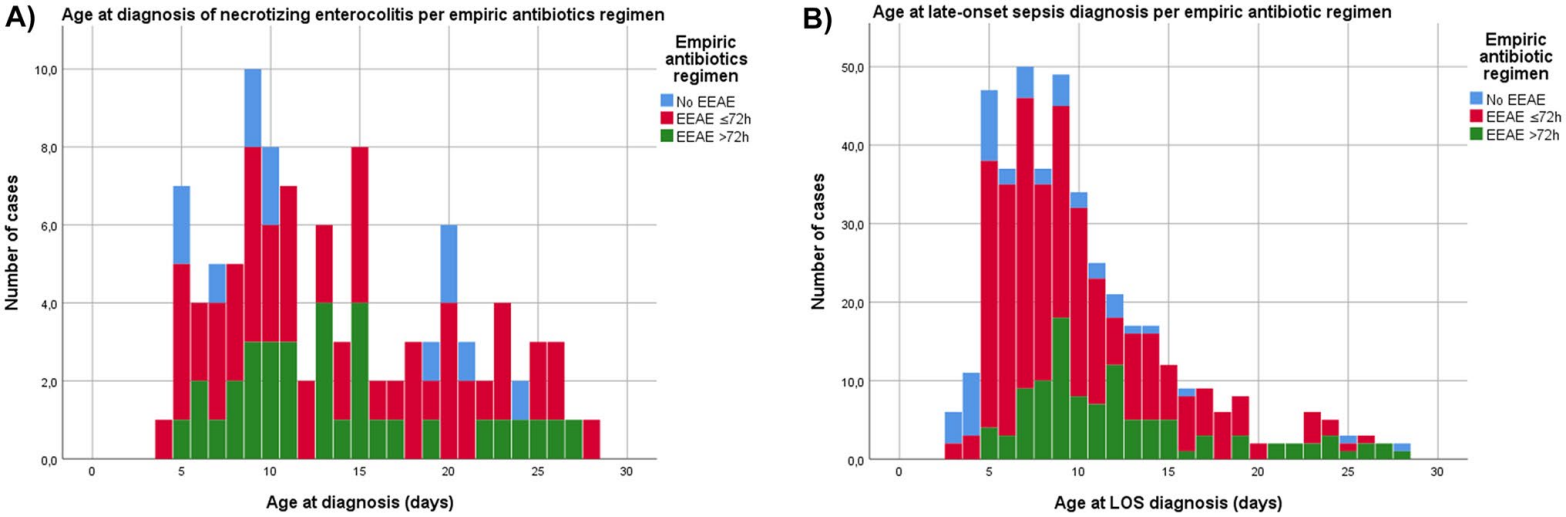
Stacked bar chart of incidence of (**A**) NEC and (**B**) LOS in the first 28 days of life, by EEAE category. EEAE, early empiric antibiotic exposure; LOS, late-onset sepsis, NEC, necrotizing enterocolitis

In the first 28 days of life, NEC occurred in 107 infants (8.4%), of whom 40 needed surgical intervention. LOS was diagnosed in 421 (33.4%) neonates, of which 192 were caused by a non-CoNS pathogen. The median age of onset of NEC was comparable between EEAE groups, while LOS occurred at a later age with increasing EEAE duration (Fig. 2A and B, resp., Table 1). Incidence of NEC and LOS by EEAE duration are represented graphically in Supplementary Fig. S1A–C and D–E, resp.

When corrected for confounding factors, the odds of NEC were lower in infants with any EEAE, compared to no EEAE (aOR 0.47; 95%CI 0.23–0.96; *p* = 0.04). Short (≤ 72 h) EEAE was associated with lower odds of developing NEC, compared to both no EEAE (aOR 0.39; 95%CI 0.19–0.80; *p* = 0.01) and prolonged (> 72 h) EEAE (aOR 0.58; 95%CI 0.35–0.96; *p* = 0.03) (Table 3). EEAE duration as a continuous variable could not be analyzed in relation to NEC incidence as the linearity assumption for logistic regression analysis was not met, regardless of data transformation or non-linear term addition.

LOS was diagnosed in 421 of the 1259 infants (33.4%). The median onset of LOS differed significantly between EEAE groups (Table 1). Table 2 demonstrates the incidences of LOS subtypes, based on causative pathogens and type of LOS. No differences were found in overall LOS incidence between infants with and without EEAE (Table 3). However, EEAE was associated with a lower incidence of non-CoNS LOS, compared to non-exposure to antibiotics (aOR 0.49; 95%CI 0.25–0.96; *p* = 0.04) (Table S3). Only prolonged EEAE, but not short EEAE, was associated with lower non-CoNS LOS incidence, compared to no EEAE (aOR 0.35; 95%CI 0.16–0.74; *p* = 0.007) (Supplementary Table S3).

**Table 2:** NEC and LOS cases shown for infants without, short (≤ 72 h), or prolonged (> 72 h) early empiric antibiotic exposure

	**NO EEAE (*N*** **= 137)**	**SHORT EEAE (*N*** **= 802)**	**PROLONGED EEAE (*N*** **= 320)**
NEC, ***N*** (%)	13 (9.5)	60 (7.4)	34 (10.6)
Surgical NEC, ***N*** (%)	4 (3)	23 (3)	13 (4)
LOS, all pathogens, ***N*** (%)	45 (32.8)	266 (33.2)	110 (34.4)
(1) CoNS LOS, ***N*** (%)	26 (19.0)	158 (19.7)	68 (21.3)
(2) All non-CoNS pathogens, ***N*** (%)	22 (16.1)	122 (15.3)	50 (15.6)
(2a) Gram-positive LOS, ***N*** (%)	14 (10.2)	49 (6.1)	21 (6.6)
(2b) Gram-negative LOS, ***N*** (%)	10 (7.3)	78 (9.7)	30 (9.4)

*AB* antibiotics, *CoNS* coagulase-negative, *EEAE* early empiric antibiotic exposure, *h* hours, *NEC* necrotizing enterocolitis, *LOS* late-onset sepsis

**Table 3:** Odds ratio of late-onset sepsis per causing pathogen between different duration of early empiric antibiotic exposure

	**OR [95%CI]**	***p*-value**	**Adjusted OR**^**a**^ **[95%CI]**	***p*-value**
NEC				
** Any EEAE vs. non**-**EEAE**	0.85 [0.46–1.57]	0.61	0.47 [0.23–0.96]	0.04*
** Short EEAE vs. no EEAE**	0.70 [0.37–1.32]	0.27	0.39 [0.19–0.80]	0.01*
** Prolonged EEAE vs. no EEAE**	1.25 [0.64–2.40]	0.52	0.65 [0.30–1.41]	0.28
** Prolonged EEAE vs. short EEAE**	1.78 [1.15–2.75]	0.01	2.56 [1.25–5.26]	0.03*
LOS, all pathogens				
** Any EEAE vs. non**-**EEAE**	1.03 (0.71–1.50)	0.88	0.78 (0.47–1.28)	0.32
** Short EEAE vs. no EEAE**	1.02 (0.69–1.49)	0.94	0.83 (0.50–1.38)	0.47
** Prolonged EEAE vs. no EEAE**	1.07 (0.70–1.64)	0.75	0.62 (0.35–1.10)	0.10
** Prolonged EEAE vs. short EEAE**	1.43 (0.80–1.39)	0.70	0.75 (0.35–1.07)	0.11
** EEAE duration (days)**	0.97 (0.92–1.02)	0.19	0.90 (0.85–0.97)	0.003**

*95%CI* 95% confidence interval, *CoNS* coagulase-negative staphylococci, *EEAE* early empiric antibiotic exposure, *LOS* late-onset sepsis, *NEC* necrotizing enterocolitis, *OR* odds ratio

^*^P < 0.05; **P < 0.01

^a^Adjusted for center, mode of delivery, gender, birth weight percentile, gestational age, Apgar score 5 min, days of parenteral feeding, invasive ventilation support, and/or inotropic medication use

When antibiotic exposure was analyzed as a continuous variable (number of days of exposure), a lower LOS incidence was found for every additional day of EEAE (aOR 0.90; 95%CI 0.85–0.97; *p* = 0.003). This negative association with the duration of empirical antibiotic exposure was observed in all subcategories of LOS (Table 3; Supplementary Table S3).

Post hoc analysis was performed solely on sepsis cases diagnosed beyond the first week. As analyzed by univariate logistic regression, prolonged EEAE was associated with higher odds for LOS, compared to both short and no EEAE. When corrected for confounding factors, this association could not be observed (Supplementary Table S4).

## Discussion/conclusion

The continuation of early empiric antibiotics despite negative blood culture results, and its effect on short-term outcomes, is debated [5, 7–9]. In this prospective multicenter cohort study, we observed that the vast majority of preterm infants are empirically exposed to antibiotics directly after birth. In about one-quarter of infants, antibiotics were continued empirically beyond 72 h, despite negative cultures. Infants with prolonged EEAE were of lower gestational age and were more often intubated, receiving inotropic medication and had higher CRP values in the first 72 h of life. They, however, had lower adjusted odds of developing LOS, compared to infants without EEAE. The group without EEAE, moreover, had higher adjusted odds of developing NEC, relative to the short EEAE group, but similar adjusted odds of NEC compared to infants with prolonged EEAE.

Similar to our findings, several studies have reported an increased risk for NEC with prolonged EEAE, compared to short EEAE [22–24]. On the contrary, the recent NEOMUNE study including 2831 VLBW infants did not demonstrate a significant difference in NEC incidence in the short antibiotic exposure (≤ 72 h) group versus the prolonged exposure (> 72 h) group: 4.3% vs. 3.7% [7]. However, they did report a lower NEC incidence (3.9%) following any early antibiotic exposure in comparison to non-exposed infants (9%) (OR 0.25, 95% 0.12–0.47, *p* < 0.001). Notably, the study population consisted of over 90% of infants receiving antibiotic treatment, of whom the majority received prolonged antibiotic treatment (> 72 h), as opposed to our cohort, in which a short course was more common. Moreover, there was a disproportionally large amount of infants born small for gestational age (SGA) and/or by caesarean section in the group of infants without EEAE, both of which are known risk factors for NEC [25]. Even though the outcome was statistically corrected for this potential confounding by indication, residual confounding may still be present. This limitation could not be avoided in our current study.

Other studies including sufficiently large groups of preterm infants not exposed to antibiotics are scarce, but our findings are further corroborated by experimental studies on preterm piglets, showing that no EEAE was associated with a higher incidence of NEC compared to EEAE [8]. EEAE resulted in increased mucosal integrity and decreased inflammatory responses, suggesting potential protective mechanisms of early antibiotics exposure on the preterm gut through immune modulation related to early gut microbiota colonization [8]. It is hypothesized that this protective mechanism could result from a delay in intestinal colonization with potential pathogens [7]. Because of this delayed colonization of pathogenic bacteria, the intestinal immune defense system might be stimulated towards postnatal adaptation [8, 9]. However, this potential beneficial effect might be negated by prolonged EEAE, as this might provoke NEC by perturbed microbial colonization [26].

One small RCT including 22 preterm infants supports the hypothesis of a protective role of short EEAE, as a more favorable microbial composition was found in infants who were randomized to 48 h of antibiotic treatment versus no EEAE [27]. Kim et al. [27] reported an increased abundance of *Actinobacteriota (*formerly *Actinobacteria)*, which was largely contributed by *Bifidobacteriaceae*, in the EEAE group, a family previously associated with a decreased risk of NEC [28]. Notably, increased *Actinobacteriota* have also been associated with NEC in other studies, however in combination with significantly decreased abundance of *Bifidobacteriaceae*. The REASON trial, a small RCT comparing a short course of antibiotics to no antibiotics, did not show a difference in microbiota between the treatment and control arm and concluded that the difference in microbiota was largely attributable to feeding type [29].

The potential protective role of EEAE for LOS that is suggested by our results, and those of el Manouni el Hassani et al. [11], is not supported by current literature on humans. Kuppala et al. [21], e.g., reported a positive association between every additional day of antibiotic exposure and LOS incidence in a preterm cohort. Their study design, however, differed in terms of follow-up period—120 days, compared to 28 days in the current study—and in terms of the study population—infants developing LOS in the first week of life were excluded by the research group [21]. In the current study, a post hoc analysis was performed excluding sepsis cases with onset < 7 days. In line with Kuppala et al.’s [21] results, unadjusted odds for LOS were lower for the non-EEAE group, compared to the prolonged EEAE group. After adjustments for confounding factors, there was no association between the duration of EEAE and LOS incidence. In our opinion, the exclusion of infants developing LOS in the first week of life might be subjected to bias, especially given that more than half of the infants who developed LOS in our non-EEAE group were diagnosed within the first week of life (median age of LOS onset 6 days). As the median age of LOS onset in the short and prolonged EEAE group was 9 and 11 days, respectively, exclusion of all LOS cases would proportionately exclude more infants with LOS in the non-EEAE group, thus underestimating LOS onset in this group. This could, however, not entirely explain the difference in results as two larger studies including 587 and 4039 infants, respectively, which did include early LOS cases during the first week of life also found a higher LOS incidence with increasing antibiotics administration [14, 30].

Although both NEC and (non-CoNS) LOS are preceded by intestinal dysbiosis [31, 32], the contrast between NEC and LOS incidence in association with EEAE might suggest different pathophysiology regarding gut microbiota-related immune responses. Despite the fact that antibiotic administration could stimulate immune maturation [33], this might not be equally relevant for different diseases and should further be explored.

The current observational study has several strengths*,* including the multicenter design, the large cohort size, and the prospective collection of detailed data on daily basis, allowing adjustment for relevant clinical and demographic factors. This also allowed us to study NEC and LOS separately and not as a combined outcome as was previously done in some studies [14, 34]. The categorization of participants based on antibiotic duration allowed us to identify non-linear associations between the duration of antibiotic exposure and NEC.

This study has several limitations, next to those characteristic of observational studies. Despite that several differences in baseline characteristics were corrected for in the multivariate analysis, there remains a risk of residual confounding of unidentified factors. Furthermore, obstetrical data could not be accessed, missing data on pre-eclampsia, umbilical cord blood flow, and intrapartum antibiotic treatment, potentially leading to underestimation of the infants’ antibiotic exposure. Registration was discontinued after the 28^th^ day of life, which could have led to missing some LOS cases. As the first LOS episode usually occurs within the first weeks of life, we hypothesized that the number of missed cases would be limited [35].

Further research on EEAE and health effects is warranted. Future perspectives include larger RCTs aiming at unravelling the effects of EEAE in low-risk infants for EOS. For example, results from the NICU Antibiotics and Outcomes (NANO) trial (ClinicalTrials.gov identifier: NCT03997266) are needed to identify the suggested (protective) effect of empirical antibiotics for NEC and LOS and to identify the optimal duration of empirical antibiotics. The interaction of antibiotics with other factors influencing the early gut colonization and immunity should be investigated. It remains to be elucidated whether current strategies against NEC, e.g., enteral feeding with human milk and the use of probiotics, have a synergistic preventive effect when combined with (short) EEAE or whether EEAE might rather be more helpful in a subgroup receiving formula feeding [36]. Studies should additionally take a broad spectrum of potential short- and long-term adverse events into account [37, 38]. In parallel, microbiota studies, preferably by metagenomics analysis, should be performed in infants receiving different lengths of empirical antibiotics to assess short- and long-term effects on intestinal colonization. In the future, these insights could allow for targeted microbiota-based preventive strategies in an optimally selected population and time window for improving the development of the immature gut [9].

Despite our findings, we believe that providing more antibiotics than currently advised, e.g., a standard short-term administration of empiric antibiotics (48–72 h) instead of watchful waiting without antibiotics in case of low risk of early-onset sepsis, should not be advised. First, the plethora of potential antibiotic-related adverse events, such as increased antibiotic resistance and other short- and long-term effects, should be further investigated [5, 39]. Current guidelines on antibiotic stewardship should be followed until results on RCTs assessing the effects of EEAE, such as the abovementioned NANO trial, are published. Empirical antibiotics should only be started when there is substantial suspicion or high risk of EOS and discontinued as soon as deemed safe (in absence of positive blood culture and reassuring clinical picture).

In conclusion, in this multicenter cohort, almost 90% of preterm infants with negative postnatal blood cultures was exposed to empirical antibiotics for suspicion of EOS. Twenty-five percent had prolonged (> 72 h) empirical exposure. A short (≤ 72 h) empirical course of antibiotics was associated with a decreased risk for NEC compared to no antibiotics and a prolonged antibiotic course. On the other hand, prolonged EEAE was associated with a decreased risk for LOS in the first 28 days of life, compared to no antibiotics. Potential antibiotic-induced changes in microbiome composition and function and their association with NEC and LOS development should be explored in future studies.

## Supplementary Information

Below is the link to the electronic supplementary material.

Supplementary file1 (DOCX 257 KB)

## Data Availability

The data used in the current study are not publicly available but are available upon reasonable request.

## Abbreviations

AB: Antibiotics
aOR: Adjusted odds ratio
CI: Confidence interval
CoNS: Coagulase-negative staphylococcus
CRP: C-reactive protein
DM: Donor milk
EEAE: Early empiric antibiotic exposure: antibiotics initiated in the first 72 h of life for the suspicion of early-onset sepsis
EOS: Early-onset sepsis
FM: Formula milk
HM: Human milk (mother’s own milk or donor milk)
LOS: Late-onset sepsis
MM: Mother’s milk
NEC: Necrotizing enterocolitis
NICU: Neonatal intensive care unit
OR: Odds ratio
SD: Standard deviation
SGA: Small for gestational age
SPSS: Statistical Package for Social Sciences
VLBW: Very low birthweight

## Footnotes

1 Netherlands: Amalia Children’s Hospital, Radboud; Amalia Children’s Center, Zwolle; Beatrix Children’s Hospital, Groningen; Emma Children’s Hospital (location AMC & location Vumc), Amsterdam; Maastricht University Medical Center, Maastricht; Máxima Medical Center, Veldhoven; Wilhelmina Children’s Hospital, Utrecht.

2 Belgium: University Hospitals Leuven, Leuven.
