# Supplementary material for: Association between duration of early empiric antibiotics and necrotizing enterocolitis and late-onset sepsis in preterm infants: a multicenter cohort study
Source: Eur J Pediatr. 2022 Aug 4;181(10):3715–24. doi: 10.1007/s00431-022-04579-5 (PMC9508214; doi:10.1007/s00431-022-04579-5)
Supplement: Supplementary file 1 — Supplementary file1 (DOCX 257 KB) [file 431_2022_4579_MOESM1_ESM.docx]

***Supplementary file***

***Association between duration of early empiric antibiotics and necrotizing enterocolitis and late-onset sepsis in preterm infants: a multicenter cohort study***

Index

[Table S1 2](#_Toc107568948)

[Table S2 3](#_Toc107568949)

[Table S3 4](#_Toc107568950)

[Table S4 5](#_Toc107568951)

[Figure S1 6](#_Toc107568952)

## Table S1

| **Table S1.** Level of care, antibiotic protocol for EOS and inclusions per EEAE duration per participating center. | | | | | |
| --- | --- | --- | --- | --- | --- |
| Inclusion Center | Level of care | Antibiotic for suspicion of EOS | No EEAE (n, %) | Short (≤72h) EEAE (n, %) | Prolonged (>72h) EEAE (n, %) |
| 1 | Level IV NICU | Benzylpenicillin + gentamicin | 23 (10) | 166 (71) | 45 (19) |
| 2 | Level IV NICU | Benzylpenicillin + amikacin | 16 (8) | 120 (63) | 56 (29) |
| 3 | Level III NICU | Amoxicillin + gentamicin | 49 (21) | 137 (60) | 43 (19) |
| 4 | Level IV NICU | Amoxicillin + gentamicin | 8 (12) | 46 (67) | 15 (22) |
| 5 | Level III NICU | Amoxicillin + ceftazidim | 10 (7) | 84 (61) | 43 (31) |
| 6 | Level IV NICU | Amoxicillin + amikacin | 12 (11) | 70 (65) | 26 (24) |
| 7 | Level IV NICU | Amoxicillin + gentamicin | 6 (8) | 45 (57) | 28 (35) |
| 8 | Level IV NICU | Amoxicillin – clavulanic acid + gentamicin | 9 (5) | 113 (68) | 44 (27) |
| 9 | Level IV NICU | Benzylpenicillin + gentamicin | 4 (9) | 21 (47) | 20 (44) |
| EOS, early-onset sepsis; EEAE, early empiric antibiotic exposure; NICU neonatal intensive care unit | | | | | |

| **Table S2.** Definitions and classification of demographics | |
| --- | --- |
| Feeding practice | |
| Formula feeding | Enteral feeding volume consisting of 50-100% formula milk |
| Full human milk feeding | Enteral feeding volume consisting of 80-100% human milk (own mother’s or donor milk) |
| Combined feeding | Enteral feeding volume consisting of <50% formula milk AND <80% human milk |
| Days of parenteral feeding | Total number of postnatal days until either 120 ml/kg of enteral feeding and/or two days without parenteral feeding (amino acids and/or lipids) was reached. |
| Late-onset sepsis (LOS) | |
| Culture-proven late-onset sepsis (LOS) | A clinical suspicion of sepsis, as reported by the treating physician, combined with a positive blood culture after the third day of life (≥72h). |
| Contaminated (CoNS) blood culture | - - - Reported as such by the treating physician, followed by immediate antibiotics cessation     - AND/OR     - CoNS-positive culture with remaining low C-reactive protein (CRP) levels (<10 mg/L)     - AND/OR     - CoNS was involved in a polymicrobial culture |
| LOS pathogen classification | 1. Coagulase negative Staphylococci (CoNS) 2. non-CoNS    1. Gram-negative pathogens    2. Gram-positive pathogens |
| Other demographics | |
| Small for gestational age (SGA) | Birthweight <10th percentile, according to the Fenton birthweight calculator [1] |
| [1] Fenton, T.R. and J.H. Kim, A systematic review and meta-analysis to revise the Fenton growth chart for preterm infants. BMC Pediatr, 2013. **13**: p. 59. | |

## Table S2

| **Table S3**. Odds ratio of late-onset sepsis per causing pathogen between different duration of early empiric antibiotic exposure | | | | | |
| --- | --- | --- | --- | --- | --- |
| Analysis | | OR [95%CI] | p-value | Adjusted OR^a^  [95%CI] | p-value |
| 1) CoNS LOS | | | | | |
|  | Any EEAE vs. non EEAE | 1.07 [0.68-1.70] | 0.77 | 1.04 [0.56-1.95] | 0.89 |
|  | Short EEAE vs. no EEAE | 1.04 [0.65-1.67] | 0.86 | 1.12 [0.60-2.09] | 0.73 |
|  | Prolonged EEAE vs. no EEAE | 1.15 [0.69-1.92] | 0.60 | 0.89 [0.44-1.78] | 0.73 |
|  | Prolonged EEAE vs. short EEAE | 1.10 [0.79-1.52] | 0.57 | 0.79 [0.53-1.19] | 0.27 |
|  | EEAE duration (days) | 0.96 [0.90-1.02] | 0.15 | 0.92 [0.86-1.00] | 0.04* |
| 2) All non-CoNS pathogens | | | | | |
|  | Any EEAE vs. non EEAE | 0.96 [0.59-1.58] | 0.89 | 0.49 [0.25-0.96] | 0.04* |
|  | Short EEAE vs. no EEAE | 0.95 [0.57-1.58] | 0.85 | 0.54 [0.28-1.05] | 0.07 |
|  | Prolonged EEAE vs. no EEAE | 1.00 [0.57-1.74] | 0.99 | 0.35 [0.16-0.74] | 0.01** |
|  | Prolonged EEAE vs. short EEAE | 1.05 [0.73-1.51] | 0.81 | 0.64 [0.39-1.06] | 0.08 |
|  | EEAE duration (days) | 0.99 [0.93-1.05] | 0.70 | 0.88 [0.80-0.96] | 0.01** |
| 2a) Gram positive LOS | | | | | |
|  | Any EEAE vs. non EEAE | 0.62 [0.33-1.14] | 0.12 | 0.38 [0.17-0.85] | 0.02* |
|  | Short EEAE vs. no EEAE | 0.60 [0.32-1.13] | 0.12 | 0.43 [0.19-0.97] | 0.04* |
|  | Prolonged EEAE vs. no EEAE | 0.66 [0.321.35] | 0.25 | 0.27 [0.10-0.71] | 0.01** |
|  | Prolonged EEAE vs. short EEAE | 1.09 [0.64-1.87] | 0.74 | 0.62 [0.31-1.26] | 0.19 |
|  | EEAE duration (days) | 0.98 [0.90-1.07] | 0.62 | 0.87 [0.77-1.00] | 0.04* |
| 2b) Gram negative LOS | | | | | |
|  | Any EEAE vs. non EEAE | 1.33 [0.67-2.64] | 0.41 | 0.56 [0.22-1.42] | 0.22 |
|  | Short EEAE vs. no EEAE | 1.34 [0.67-2.68] | 0.41 | 0.62 [0.24-1.58] | 0.32 |
|  | Prolonged EEAE vs. no EEAE | 1.31 [0.61-2.80] | 0.48 | 0.36 [0.12-1.01] | 0.05 |
|  | Prolonged EEAE vs. short EEAE | 0.98 [0.63-1.54] | 0.94 | 0.57 [0.30-1.09] | 0.09 |
|  | EEAE duration (days) | 0.98 [0.91-1.06] | 0.59 | 0.85 [0.75-0.97] | 0.02* |
| * P<0.05; **P<0.01  ^a^ Adjusted for Center, Mode of delivery, Gender, Birth weight percentile, Gestational age, Apgar score 5 min, days of parenteral feeding, invasive ventilation support and/or inotropic medication use  95%CI, 95% confidence interval; CoNS: coagulase-negative staphylococci; EEAE: early empiric antibiotic exposure; LOS: late-onset sepsis; NEC: necrotizing enterocolitis; OR: odds ratio. Data is summarized as odds ratio (95% confidence interval). | | | | | |

## Table S3

| **Table S4**. Odds ratio of late-onset sepsis per causing pathogen between different duration of early empiric antibiotic exposure | | | | | |
| --- | --- | --- | --- | --- | --- |
| Analysis | | OR [95%CI] | p-value | Adjusted OR^a^  [95%CI] | p-value |
| 1) All LOS with onset at age ≥7 days (n=323, 28%) | | | | | |
|  | Any EEAE vs. non EEAE | 1.50 [0.99-2.57] | 0.06 | 1.04 [0.60-1.80] | 0.89 |
|  | Short EEAE vs. no EEAE | 1.46 [0.90-2.37] | 0.13 | 0.97 [0.56-1.68] | 0.92 |
|  | Prolonged EEAE vs. no EEAE | 1.94 [1.16-3.25] | 0.01* | 0.93 [0.52-1.68] | 0.82 |
|  | Prolonged EEAE vs. short EEAE | 1.33 [1.00-1.75] | 0.05 | 0.96 [0.69-1.33] | 0.80 |
|  | EEAE duration (days) | 1.03 [0.98-1.08] | 0.22 | 0.97 [0.91-1.03] | 0.29 |
| 2) All non-CoNS pathogens with onset at age ≥7 days (n=151, 13%) | | | | | |
|  | Any EEAE vs. non EEAE | 1.93 [0.95-3.92] | 0.07 | 1.11 [0.48-2.53] | 0.81 |
|  | Short EEAE vs. no EEAE | 1.82 [0.81-3.73] | 0.10 | 1.18 [0.51-2.72] | 0.70 |
|  | Prolonged EEAE vs. no EEAE | 2.22 [1.04-4.72] | 0.04* | 0.94 [0.38-2.30] | 0.89 |
|  | Prolonged EEAE vs. short EEAE | 1.22 [0.82-1.79] | 0.32 | 0.79 [0.50-1.27] | 0.34 |
|  | EEAE duration (days) | 1.05 [0.99-1.12] | 0.11 | 0.99 [0.91-1.07] | 0.71 |
| * P<0.05  ^a^ Adjusted for Center, Mode of delivery, Gender, Birth weight percentile, Gestational age, Apgar score 5 min, days of parenteral feeding, invasive ventilation support and/or inotropic medication use  95%CI, 95% confidence interval; CoNS: coagulase-negative staphylococci; EEAE: early empiric antibiotic exposure; LOS: late-onset sepsis; NEC: necrotizing enterocolitis; OR: odds ratio. | | | | | |

## Table S4

## Figure S1


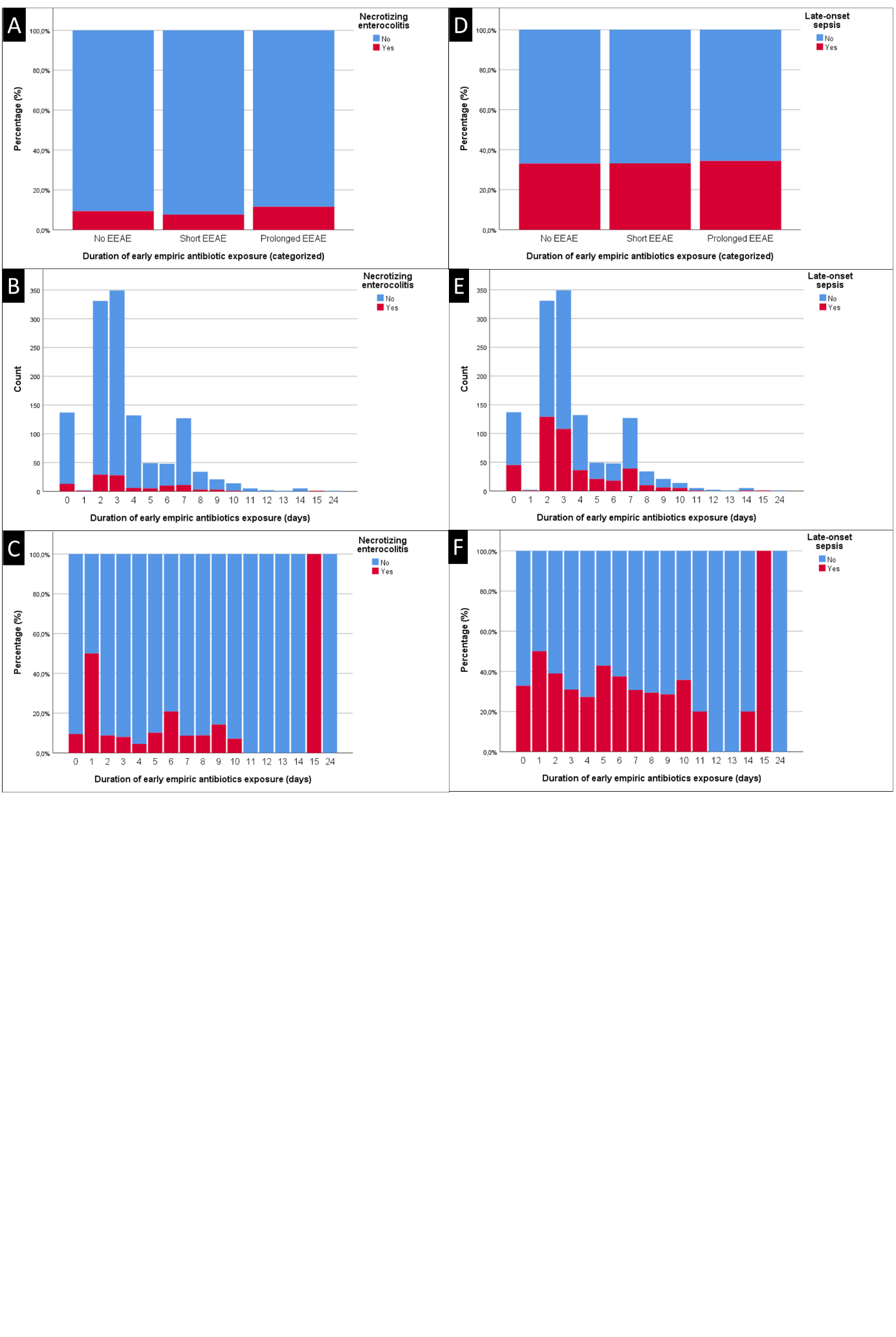


Figure S1. Incidence of necrotizing enterocolitis (A-C) and late-onset sepsis (D-E) by duration of early empiric antibiotics administration. A+D) Relative incidence (percentage) of cases per category of EEAE; B+E) Absolute incidence per amount of days of EEAE; C+F) Relative incidence per amount of days of EEAE.

*Data used for this graph are not adjusted for confounding factors and trends might differ from trends observed* *by performing multivariate regression analysis*.

*EEAE, early empiric antibiotics exposure*
